# Exposure to metals and morbidity at eight years follow-up in women of childbearing age

**DOI:** 10.1038/s41598-021-90904-1

**Published:** 2021-06-01

**Authors:** Isabella Karakis, Yael Baumfeld, Daniella Landau, Roni Gat, Nofar Shemesh, Maayan Yitshak-Sade, Ofir Tirosh, Batia Sarov, Lena Novack

**Affiliations:** 1 Environmental Epidemiology, Ministry of Health, Jerusalem, Israel; 2 Obstetric and Gynecology, Soroka University Medical Center, Beer-Sheva, Israel; 3 Neonatology Department, Soroka University Medical Center, Beer-Sheva, Israel; 4 Negev Environmental Health Research Institute, Soroka University Medical Center, Beer-Sheva, Israel; 5 Faculty of Health Sciences, Ben-Gurion University of the Negev, Beer-Sheva, Israel; 6 Department of Clinical Biochemistry and Pharmacology, Ben-Gurion University of the Negev, Beer-Sheva, Israel; 7 The Fredy and Nadine Herrmann Institute of Earth Sciences, The Hebrew University in Jerusalem, Jerusalem, Israel; 8 Icahn School of Medicine At Mount Sinai, Department of Environmental Medicine and Public Health, New York, NY USA; 9 Department of Public Health, Ben-Gurion University of the Negev, Beer-Sheva, Israel; 10 Clinical Research Center, Soroka University Medical Center, POB 651, 84101 Beer-Sheva, Israel

**Keywords:** Environmental sciences, Diseases

## Abstract

This exploratory study was aimed to investigate the link between toxic metal content in women’s urine and their morbidity 2 years before and 6 years after the test. Concentrations of 25 metals in urine were analyzed for 111 pregnant women collected prior to delivery. All women were of Arab-Bedouin origin. Information on primary care and hospital visits during the study period was obtained. In a Poisson regression model, a health outcome was regressed over metal exposure and other factors. A Weighted Quantile Sum Regression (WQS) approach was used to indicate metals dominating in their possible impact on women's morbidity. Obesity was the most frequently diagnosed condition in this population (27.9%). Diagnoses in a neurological category accounted for 36.0%, asthma or respiratory—25.2%, psychiatric—12.6%, cardiovascular—14.4% and cancer or benign growth—for 13.5%. Based on WQS analysis, cancer and benign growth were mostly attributed to the increased levels of cadmium, cardiovascular outcomes were linked with lead, and obesity was found associated with elevated levels of nickel. Hematological, neurological and respiratory outcomes were attributed to multiple non-essential metals. The health and exposure profile of women in the study warrants a periodic biomonitoring in attempt to identify and reduce exposure to potentially dangerous elements.

## Introduction

It has been established by scientific communities that environmental pollution poses a threat to public health, causing about seven million deaths worldwide every year, as per WHO estimates^1^. Since all countries without exclusion are subjected to certain levels of pollution, its potential impact is relevant to all. For this reason the range of morbidities related to environment has been coined as "civilization" diseases, i.e. affecting the world in its entirety^2,3^ The most frequent diseases related to environment are malignancies, cardiovascular morbidity, certain brain disorders, fetal complications and pulmonary, endocrine, and immune disorders.

In spite of the link between environment and health recognized by the scientists^4–6^, there is no consensus on the *pathophysiological pathway* between the two^7–10^. To elucidate the way pollution might impact a human body, one has to estimate an extent of internal exposure to pollution biomarkers usually provided by human biomonitoring (HBM) techniques. Metals detected in urine represent classical biomarkers of environmental exposure, nevertheless, investigations of metabolomic profiles of population are rare^11^, and therefore their potential impact is largely unknown.

Nonetheless, in some instances, an exposure to a certain chemical can be linked to a certain disease, or in other words, there are cases in which an exposure to a chemical is relatively specific to a morbidity, a prerequisite for establishing causality. There are few examples of these links, as follows.

- In a study by Karakis et al., newborns whose mothers were exposed to aluminum (Al) were at higher risk of minor malformations diagnosed at birth^12^. Mothers exposed to arsenic (As) were more likely to develop gestational diabetes^12^.
- Among subjects from the National Health and Nutrition Examination Survey (NHANES), cadmium (Cd) was reported related to cardiovascular disease; tungsten (W) and uranium (U) were linked to asthma; molybdenum (Mo) was associated with hepatotoxicity; lead (Pb)—with obesity; and cobalt (Co) and cesium (Cs)—with reduced visual acuity^13^.
- Cd, nickel (Ni), Al, arsenic (As) and beryllium (Be) have been defined as carcinogenic to humans (group 1) by International Agency for Research on Cancer^14^.
- Cd and lead (Pb) have been linked to neurological morbidity, e.g. multiple sclerosis^15^.

In all the instances above, using the HBM methodology was essential to the studies' conclusions.

Although exposure to pollution is universal, its main burden falls on the low-income populations, often residing close to roads or industrial zones, and therefore especially prone for development of health disorders. Monitoring of pollution in these populations is crucial for primary prevention of diseases, especially in case of well-established chemical-disease links.

The current analysis was focused at the population of women of childbearing age of Arab-Bedouin origin residing in the southern Israel. This population is leading a semi nomadic lifestyle, while approximately half of them lives in temporary tents or shacks, frequently using open fire for cooking or heating. The majority of the male population is smoking^12,16,17^. This way of life coupled with low socioeconomic status makes the Arab-Bedouin minority potentially susceptible to pollution and possibly contributes to the high morbidity rates^18,19^. Periodic monitoring of an exposure profile of women of childbearing age is essential for primary prevention of morbidity in both, their offspring and women themselves.

This exploratory study was aimed to investigate the association between toxic metal content in women’s urine samples and their morbidity status defined 2 years before and 6 years after the test.

## Results

In all, out of 1823 women enrolled in the study cohort, 1437 met the inclusion criteria and 111 of them had a urine sample available for the analysis. The women were on average 28.1 ± 6.3 years old, and one third of them had their sixth delivery or more (Table 1). Reports on living in a temporary tent of shack and cooking on open fire were frequent (29.5% and 74.6%, respectively) and most of them reported being married to their cousins (72.2%). Judging by maternal age, parity, gestational age at birth, infant weight, gender, living in a temporary shack and cooking on open fire, this sample was not statistically different from the main cohort, except for consanguineous marriage reported for 49.7% of women in the original cohort (data not shown).

**Table 1:** Demographic characteristics of the study population.

Subjects’ characteristics	Study sample (N = 111 newborns) (N = 111 deliveries)
**Demographical factors**	
Maternal age, years	
Median	26.9
Min; Max	18.4; 41.7
Parity	
1	27.3 (30/110)
2–5	42.7 (47/110)
6 +	30.0 (33/110)
Residing in a temporary shack/tent, % (n/N)	29.5 (18/61)
Cooking on open fire, % (n/N)	74.6 (41/55)
Consanguineous marriage, % (n/N)	72.2 (39/54)
**Morbidities**	
Hematological, % (n/N)	5.4 (6/111)
Cancer/Benign, % (n/N)	13.5 (15/111)
Cardiovascular, % (n/N)	14.4 (16/111)
Psychiatric, % (n/N)	12.6 (14/111)
Obesity, % (n/N)	27.9 (31/111)
Diabetes Mellitus, % (n/N)	8.1 (9/111)
Neurological, % (n/N)	36.0 (40/111)
Asthma/Respiratory % (n/N)	25.2 (28/111)

Women in the sub-sample with metals' evaluation were similar to the main cohort in the distribution of morbidity types. Obesity was the most frequently diagnosed condition in this population, prevalent in 27.9% of the women. The rest of the diagnoses were grouped into neurological category accounting for 36.0%, asthma or respiratory—25.2%, psychiatric—12.6%, cardiovascular—14.4% and cancer or benign growth—for 13.5% (Table 1).

**Figure 1:**
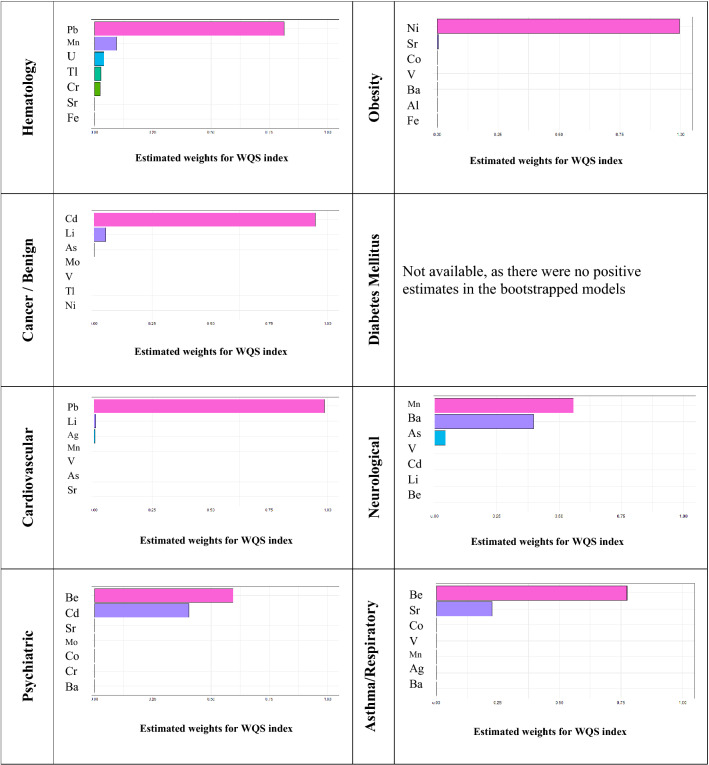
Metals' burden on morbidity. Results of Weighted Quantile Sum Regression analysis.

The majority of the metals were associated between each other, although the magnitude of correlation (rho) varied significantly between the metals. Focusing on associations between non-essential metals alone, the metals thallium (Tl)-Mo-Cd, lithium (Li)- selenium (Se)-Co–Ni and iron (Fe)-silver (Ag) appeared to be strongly and positively linked with rho > 0.60 (Fig. 1, supplementary material).

### Association of morbidities with individual metals' concentrations

The associations between metals' concentrations and morbidities were mostly in the positive direction after adjusting to the subjects' age (Table 2). Below is the summary of findings including associations with borderline significance, i.e. p-value within the range 0.05–0.10.

- *Hematological* outcomes were linked to higher concentrations of chromium (Cr) (Prevalence Ratio (PR) = 2.09, *p*-value = 0.073).
- *Cancerous or benign growths* were linked to Cd and As (PR = 1.75, *p*-value = 0.020 and PR = 1.43, *p*-value = 0.039, respectively).
- *Cardiovascular* endpoints were more likely to be diagnosed in women with higher manganese (Mn) (PR = 1.41, *p*-value = 0.027) and Ag (PR = 1.74, *p*-value = 0.001).
- *Obesity* was linked to Al (PR = 1.39, *p*-value = 0.026), vanadium (V) (PR = 1.22, *p*-value = 0.097) and Fe (PR = 1.25, *p*-value = 0.069), and also biometals, e.g. potassium (K) (PR = 1.36, p-value = 0.027), Ca (PR = 1.26, *p*-value = 0.091) and copper (Cu) (PR = 1.36, *p*-value = 0.032).
- *Neurological* morbidity was linked to barium (Ba) (PR = 1.20, *p*-value = 0.101) and As (PR = 1.21, *p*-value = 0.074).

**Table 2:** Association between internal dose of metals in quartiles and presence of a clinical outcome at follow-up expressed as Prevalence Ratio (PR), (*p*-value)^1,2^.

	Metal3 (n = 111)	Geometric mean	95% CI	Hematology (n = 6) vs none (n = 105)	Cancer/benign (n = 15) vs none (n = 96)	Cardiovascular (n = 16) vs none (n = 95)	Psychiatric (n = 14) vs none (n = 97)	Obesity (n = 31) vs none (n = 80)	DM (n = 9) vs none (n = 102)	Neurological (n = 40) vs none (n = 71)	Asthma/respiratory (n = 28) vs none (n = 83)
Biometals	Na, ppm	1629.3	969.3; 2738.7	1.11 (.774)	1.48 (.129)	0.96 (.844)	0.71 (.082)	1.23 (.140)	0.93 (.824)	1.18 (.126)	0.97 (.816)
	K, ppm	998.9	604.8;649.6	0.64 (.774)	1.16 (.463)	1.07 (.734)	0.91 (.677)	1.36 (.027)	1.24 (.439)	1.18 (.142)	1.00 (.975)
	Mg, ppm	15.2	9.4; 24.4	1.13 (.603)	1.20 (.398)	1.15 (.470)	1.00 (.997)	1.23 (.132)	1.06 (.823)	0.99 (.933)	0.94 (.671)
	Ca, ppm	19.7	12.3; 31.5	1.11 (.752)	1.30 (.231)	1.02 (.939)	1.21 (.327)	1.26 (.091)	1.04 (.874)	1.07 (.549)	1.26 (.097)
	Se, ppb	19.6	13.7; 27.9	0.85 (.644)	1.23 (.374)	1.31 (.174)	1.15 (.522)	1.23 (.142)	1.03 (.913)	1.00 (.965)	0.97 (.836)
	Zn, ppb	146.5	93.6; 229.3	1.07 (.786)	1.27 (.261)	1.28 (.115)	1.11 (.653)	1.16 (.306)	1.19 (.548)	1.13 (.267)	0.91 (.508)
	Cu, ppb	12.1	8.6; 17.1	0.73 (.378)	1.29 (.200)	1.06 (.805)	1.01 (.976)	1.36 (.032)	0.99 (.970)	1.16 (.197)	0.88 (.334)
Non-essential metals	Li, ppb4	6.6	4.6; 9.4	0.89 (.706)	1.36 (.169)	1.33 (.247)	–	1.02 (.908)	1.25 (.529)	1.10 (.442)	0.72 (.026)
	Co, ppb	0.9	0.7; 1.2	0.87 (.638)	0.92 (.653)	1.09 (.636)	1.11 (.575)	1.20 (.177)	0.94 (.824)	1.03 (.783)	0.99 (.943)
	Ni, ppb	1.2	1.0; 1.6	0.97 (.931)	1.47 (.108)	1.07 (.730)	1.02 (.906)	1.27 (.087)	0.99 (.982)	1.05 (.672)	0.92 (.509)
	Tl, ppb	0.04	0.0; 0.1	1.72 (.023)	1.47 (.072)	1.11 (.613)	0.79 (.201)	1.05 (.717)	0.95 (.837)	0.93 (.463)	0.87 (.292)
	Al, ppb	6.1	3.8; 9.9	1.30 (.497)	1.12 (.608)	1.08 (.700)	0.76 (.247)	1.39 (.026)	1.04 (.919)	0.91 (.387)	0.89 (.437)
	Cr, ppb	0.6	0.4; 0.9	2.09 (.073)	1.07 (.680)	1.10 (.678)	1.44 (.086)	0.99 (.921)	0.70 (.299)	1.06 (.575)	0.94 (.697)
	Sr, ppb	72.7	48.3; 109.7	1.46 (.209)	1.16 (.471)	1.24 (.209)	1.21 (.329)	1.19 (.213)	0.81 (.482)	1.07 (.563)	1.15 (.331)
	Ba, ppb	1.3	0.9; 1.9	1.24 (.434)	0.91 (.668)	1.15 (.488)	1.34 (.218)	1.19 (.190)	0.65 (.181)	1.20 (.101)	1.13 (.339)
	Cd, ppb	0.2	0.2; 0.3	1.24 (.517)	1.75 (.020)	1.05 (.802)	1.05 (.801)	1.05 (.742)	1.12 (.682)	1.08 (.448)	0.90 (.448)
	Be, ppb	0.1	0.1; 0.2	0.81 (.434)	0.82 (.295)	0.74 (.120)	1.26 (.321)	1.18 (.180)	1.00 (.988)	1.11 (.353)	1.11 (.450)
	V, ppb	0.04	0.0; 0.1	1.13 (.666)	1.26 (.240)	1.40 (.082)	0.78 (.202)	1.22 (.097)	1.34 (.287)	1.08 (.454)	1.01 (.938)
	As, ppb	3.6	2.3; 5.5	1.31 (.438)	1.43 (.039)	1.36 (.102)	0.79 (.310)	1.06 (.633)	0.96 (.898)	1.21 (.074)	0.83 (.209)
	Fe, ppb	1.2	0.5; 2.5	1.34 (.438)	1.24 (.231)	1.21 (.470)	0.75 (.194)	1.25 (.069)	0.94 (.806)	1.06 (.567)	0.99 (.946)
	Mo, ppb	7.2	3.9; 13.6	1.15 (.668)	1.38 (.132)	1.11 (.589)	1.28 (.191)	1.05 (.724)	0.89 (.734)	1.01 (.908)	0.72 (.027)
	Mn, ppb	0.03	0.02; 0.04	1.34 (.774)	1.05 (.759)	1.41 (.027)	1.01 (.965)	0.86 (.284)	1.32 (.262)	1.09 (.333)	1.13 (.297)
	Ag, ppb	0.04	0.0; 0.1	1.12 (.710)	1.05 (.790)	1.74 (.001)	0.86 (.502)	1.08 (.494)	1.04 (.900)	1.05 (.586)	1.05 (.654)
	Pb, ppb	0.2	0.1; 0.3	1.72 (.138)	0.90 (.527)	1.38 (.084)	0.95 (.800)	1.00 (.966)	1.05 (.857)	0.86 (.127)	0.96 (.732)
	U, ppb	0.01	0.01; 0.01	1.41 (.280)	1.10 (.623)	0.91 (.668)	0.73 (.294)	0.81 (.187)	1.01 (.958)	0.88 (.364)	0.86 (.336)
Any non-essential metal in 4th quartile, % (n/N)											
in the group with disease	100.0 (6)	100.0 (15)	100.0 (16)	92.9 (13)	87.1 (27)	77.8 (7)	95.0 (38)	96.4 (27)			
in the group without disease	84.8 (89)	83.3 (80)	83.2 (79)	84.5 (82)	85.0 (68)	83.6 (88)	80.3 (57)	84.3 (70)			
p-value for comparison	0.301	0.087	0.076	0.407	0.778	0.487	0.034	0.096			
Number of non-essential metals in 4th quartile											
In the group with disease Median	4.0	5.0	5.5	4.0	5.0	5.0	4.0	3.5			
In the group without disease Median	4.0	4.0	4.0	4.0	4.0	4.0	4.0	4.0			
p-value for comparison	0.560	0.440	0.076	0.617	0.271	0.656	0.753	0.388			

^1^The table presents a Prevalence Ratio (PR) reflecting a multiplicative variation in the metals' concentrations associated with presence of a health outcome, adjusted to age. Age was statistically associated with the cardio-vascular outcome, psychiatric morbidity, obesity and diabetes mellitus.

^2^Estimates with *p*-value < 0.1 are shaded.

^3^Li values in subjects with a psychiatric diagnosis were defined as missing.

No significant associations with elevated metal content were found for *diabetes*.

Half of the women in the study had more than 4 of non-essential metals in the highest quartile. In general, subjects exposed to non-essential metals were more likely to be diagnosed by one of the study outcomes, especially evident with cardiovascular and neurological endpoints.

### Identifying metals with highest contribution

An attempt to single out the metals with the most potential impact on the morbidity was made. The weighted quantile sum (WQS) regression methodology provided with the weight of each of the metal out of the overall impact expressed in percent (Fig. 1).

Three morbidity types were strongly related to one dominating metal, i.e. cancer and benign growth—Cd, cardiovascular was associated with Pb and obesity—with Ni. Other endpoints shared their source between the elements. For instance, hematological outcome was mostly attributed to Pb, and was followed by Mn, Li, Tl and Cr. Neurological outcomes were related almost equally to Mn and Ba, but also to As. Asthmatic—respiratory outcome was linked with beryllium (Be) and strontium (Sr). Sensitivity analysis of deciles and percentiles did not impact the order of the metals' contribution to the outcomes within the first 2 metals.

Important to mention that the WQS methodology unadjusted for age and accounting for correlations between the metals was not always aligned with the findings obtained from Poisson regression approach adjusted for age. For instance, impact of Cr concentrations on hematological outcome was characterized by the highest point estimate (PR = 2.09), however the WQS approach attributed most of the burden to Pb. Likewise, the same change in weights of metals was observed for the rest of morbidities, except for cancer where exposure to Cd dominated over other elements.

## Discussion

In the current analysis, multiple links between metals in urine and morbidities were identified. Estimates of association for most of the non-essential metals with health pointed at the positive direction, whereas higher concentrations were likely to be found in women with an outcome.

### Association between metals and morbidity

The WQS regression was particularly useful for choosing the dominant metals in their impact on health, while adjusting for multiple pairwise correlations between the metals. The analysis by WQS pointed at the dominant weight of *Pb* in association with hematological and cardiovascular morbidity, *Cd—*with cancer, and *Ni—*with obesity. These morbidities were almost solely dominated by the aforementioned metals, indicating the high specificity of exposure to them.

- The link between metals' concentrations and *cardiovascular morbidity* is supported by other researchers, although not the exact list of metals used in the analysis. Thus in a large sample of 1277 pairs of patients with ischemic stroke and their matched controls, the researchers found that patients were more likely to have higher concentrations of Al, As and Cd^25^. These metals are all adversely, however not statistically linked to cardiovascular morbidity also in the current study.
- Cd is known for its *carcinogenic* effect on humans and therefore, the link observed in this analysis is well supported by other reports^26^.
- In the current analysis, almost all the metals appeared to be adversely related to *obesity*, although not always statistically meaningful. The association between obesity and Ni recorded in the study has not been reported so far. The analysis of 6–19 your old children in NHANES cohort showed a link of obesity with Ba, however, Cd, Co and Pb appeared to be a protective factor^27^. A study of adults in NHANES showed that higher levels of metals in blood or urine were associated with higher body mass index (BMI)^28^. A review by González-Casanova et al. suggests that the possible pathological pathway to this link is similar to the mechanism of endocrine disrupting chemicals^29^.
- *Hematology-related outcomes, neurological, respiratory and psychiatric* morbidities appeared to be associated with more than one dominant metal, which possibly has to do with their multi-factorial nature. Similar to other diagnoses, the list of dominant metals included only industry-related elements, like Mn, Be, Co, Ba and Sr.
- No adverse, or in fact, any association of metals with *diabetes mellitus* (DM) was found, even for As, well-known for its contribution to DM^20^. This is opposed to the researchers' expectation, as diabetes has been repeatedly shown to be related to environmental pollution. For instance, Canadian researchers linked high Glucose values with elevated persistent organic pollutants (POPs) in blood, by also indicating a dose–response relationship between the two^21^. The NHANES survey for the period of 1999–2010 pointed at Mo, antimony (Sb), W and U as potential risk factors for DM^22^. The association was established for a multivariable comparison of the highest quartile of content to the lowest with odds patios (OR) 2.76, 2.72, 2.18 and 1.46 for Mo, Sb, W and U, respectively, based on a large sample of 9,447 subjects. Furthermore, Sade et al. showed the link between pollution and DM in two population-based studies. Specifically, elevated Glucose values were recorded following exposure to Nitrogen Dioxide (NO_2_) and Sulfide Dioxide (SO_2_)^23^ and increased hemoglobin A1C (HbA1c) were reported following exposure to higher ambient levels of particulate matter^24^. As anthropogenic air pollution potentially contains metals, the link between this diagnosis and metal concentrations was well expected. The non-significant association we obtained is probably due to using a binary diagnosis of DM, and not continuous DM biomarkers, glucose or HbA1c, used by others, in addition to the small sample size in the current analysis.

### Morbidity in the study population

The current report provides a snapshot of the morbidity range in the population of women of Arab-Bedouin origin. Due to their lifestyle, this population closely resembles countries of the third world, however, the one with a direct access to modern medicine and therefore, up-to-date diagnostic procedures and high quality of medical records. High morbidity rates of most of environment-related diseases were observed, especially striking to find among relatively young women, all being under 47 years old. The close inspection of the diagnoses reveals a wide range of medical conditions that require an educated physician's judgment and less reflect patients' behavior, i.e. with diagnoses in the neurological or asthma and respiratory group. The health profile of the study population is comparable to reports from the United States with Arab population being a minority, indicating higher rates of diabetes in this population in California as compared to non-Hispanic whites^30^, as well as, hypertension, and particularly more frequent influenza and pneumonia among women in the Michigan state^31^.

The rate of obesity (close to 28%) is alarmingly high, nevertheless, consistent with the overall increase in BMI reported worldwide^32^, especially within a stratum of low socioeconomic status where it enhances the risk of chronic morbidity^33^. Ganwar et al. indicated a higher prevalence of cardiovascular morbidity among the subjects residing close by an e-waste burning site featured by high concentrations of Pb, Cu, zinc (Zn), Ni and Cr^34^. Other studies are less specific about the particular metals, and point at the possible link based on the exposures in the working environment. For instance, Bulka et al. show such association between working with metals and cardiovascular diseases^35^.

The study findings have to be treated with caution in view of its *limitations*.

- The most obvious one is its small sample size that precluded from performing an in-depth analysis of metal-related associations. Even so, the sample used in this analysis closely resembles its initial cohort and therefore, is likely to reliably represent the Arab-Bedouin women of child-bearing age in the area.
- A part of our population has been missing questionnaires due to the deliveries taking place during weekends or holidays, coupled with short hospitalization stays, when our research staff could not reach all enrolled subjects. On the other hand, enrollment at delivery room ensured the population-based approach in the study expected to attenuate a selection bias.
- The temporal association between exposure and the outcomes could not be accurately identified, the latter relying on a diagnosis usually assigned with a substantial delay from a disease onset. Exposure however, probably represents a chronic condition of the Bedouin population in the area, as it rarely changes its lifestyle or moves to another geographic location.

To conclude, the majority of chemicals are adversely associated with chronic morbidity, whereas the dominating elements being Pb, Cd, Mn, Be, Co and Ni, all industry or transport related metals. The health and exposure profile of women in the study warrants a periodic biomonitoring in attempt to identify and reduce exposure to potentially dangerous elements.

## Methods

### Study population

The current analysis explores a subset of a cohort of women of Bedouin-Arab origin enrolled between Dec 2011–Mar 2013 upon their hospitalization at delivery wards. The enrollment procedures and characteristics of the study population have been described previously^12^. Briefly, all women of Bedouin-Arab origin arriving at the obstetrics emergency department for a delivery at Soroka University Medical Center (SUMC) during the regular working hours, were approached by an Arabic-speaking interviewer and invited to participate in the study. Upon signing the informed consent form, the women's spot urine sample was collected. The main restriction in urine collection was the difficulty to obtain the sample hours and sometimes minutes before the delivery. The questionnaire on the demographical and clinical characteristics of the women was administered during the hospitalization following the delivery. In all, 1823 women were enrolled in the cohort. To be included in the current analysis, the participants had to be the members of "Clalit" health maintenance organization (HMO) (in all, 1437 women) and to have an available urine sample collected prior to birth and preserved in the freezer till the testing became available (n = 111).

### Urinary metals determination

Concentrations of 25 metals in urine samples were estimated. Samples were stored at -20^0^C prior to their testing five to six years later. An Agilent 7500 Series inductively coupled plasma mass spectrometer (ICP-MS) (Agilent Technologies, Tokyo, Japan) was used to assess the metal content of each sample. Specifically, concentrations of sodium (Na), K, magnesium (Mg), calcium (Ca), Zn, Se, Cu, Li, Co, Ni, Tl, Al, Cr, Sr, Cd, Ba, Be, V, Fe, As, Mo, Pb, Ag, Mn and U were evaluated. Testing was performed in a trace metal clean room, a laboratory in the Institute of Earth Sciences at the Hebrew University of Jerusalem. Values below the level of quantification (LOQ) were imputed by LOQ divided by the square root of two. The LOQ in all the metals was equal 0.01.

Lithium concentrations were not analyzed if they were recorded in subjects with psychiatric diagnoses.

### Outcomes definition and other clinical information

Verification of the study outcomes relied on the medical information recorded by the local hospital and HMO medical personnel during the 2 years prior to the index delivery and 6 years after. The hospitalization records were pulled from the Admission-Transfer-Discharge (ATD) database and included details on the subjects' diagnoses assigned at the Emergency Room (ER) visits, hospitalizations and outpatient clinics. HMO records captured the visits to primary physicians and specialists' clinics. No pregnancy–related diagnoses were included, aiming to focus only on the chronic morbidity.

The following categories of morbidity were verified: hematology, cancer and benign, cardio-vascular, psychiatric, obesity, diabetes mellitus, neurological and asthma or other respiratory morbidity. The complete list of diagnoses in each of the categories is presented in Table 1 (supplementary material) along with the frequencies in the study population. Women were defined as diagnosed if a diagnosis appeared at least once in the medical record throughout the entire study period.

### Statistical analysis

Metal concentrations in urine were reported as geometric means along with a 95% Confidence Intervals (CI) and compared by subjects' morbidity status using a ratio t test. Linearity assumption in the association between metals' concentrations ranked into quartiles and the health outcomes was explored visually. The correlation structure between the metals was explored using Spearman rho estimates, and presented in a correlation plot.

We analyzed the data using (a) a classical approach looking for the association between morbidities and concentrations of individual metals and (b) identifying the main contributing metals for each of the study outcomes.

- (a) *Association between individual metals and morbidity*

We used the directed acyclic graphs (DAG) technique^36^ facilitating the choice of the minimal adjustment set to be used in the analysis to avoid confounding. The minimal adjustment set generated by DAG included a socioeconomic status (SES) that followed the underlying assumption that SES is associated with both, exposure and the outcomes. However, this data generating process was not supported by the collected data. Specifically, an indicator of housing conditions, tent or shack vs a standard house, was not found associated neither with the metals' concentrations nor with the morbidities. Likewise, a standardized SES score assigned by the Israel Central Bureau of Statistics based on the subjects' residence address, was not applicable, as SES within the Bedouin population is one of the lowest in the country and is not sufficiently diverse to provide the needed effect. Furthermore, about half of the population does not have a street address, and is frequently assigned to a centroid of a nearby town, compromising the score accuracy. The associations were adjusted to maternal age, to account for diversity in outcome distribution, and by doing so, increasing the accuracy of the models.

Adjustment for individual risk factors was performed using a Poisson regression model corrected for over-dispersion. This regression was preferred over Negative Binomial regression due to its efficiency and minimal convergence problems. To ensure the robustness of the model inference, the sandwich estimator approach for standard error calculation was chosen as the most conservative option. Adjusted point estimates of an association were presented as prevalence ratio (PR), to account for the fact that diagnoses included 2 years prior to the metals' evaluation in urine that precluded estimation of relative risk.

- (b) *Identifying the main contributing metals*

Finally, the current analysis was aimed to identify the main contributing metals for each of the study outcomes by means of WQS regression. This method accounts for the correlation between the elements and regresses the weighted sum of the exposure (usually, in quantiles) on the health outcome expectation. In the analysis, the outcome was defined as binomially distributed and the metal concentrations as their original values. Different quantile formats, e.g. quintiles, deciles and percentiles, were tested as a part of the sensitivity analysis. This approach has been suggested by Nordberg et al.^37^ and recently used by Daniel et al. in an investigation of exposure to phthalate and fine-motor functions in children^38^. This methodology allows accounting for strongly correlated exposures featuring pollutants of any kind that would violate regular analysis due to collinearity. Instead of using all correlated pollutants in one regression, the WQS regression reassigns sets of weights to the pollutants' levels and summarizes them in a weighted sum of pollutants' quantiles. The set of weights best predicting the outcomes is selected and pollutants weights are described.

This methodology was applied to all non-essential metals, i.e. all the metals after excluding Na, K, Mg, Ca, Se, Zn and Cu, as suggested by other researchers^39^.

Due to the relatively small sample size and yet a large number of metals (in all 18), the number of metals tested by WQS was reduced to 7, based on the previous log-linear multivariable analysis applied separately to each metal. Specifically, 7 metals with the highest point estimates (PRs) were chosen indicating an adverse effect on the outcome (PR > 1), and after adjusting to age in a multivariable analysis. This two-staged approach was aimed to reduce over-fitting in WQS and at the same time, by optimally choosing metals with the most potential on health independently of other covariates.

The analyses were performed using SAS 9.4 and R software. The gWQS package was used for WQS regression analysis. In view of the explorative nature of this analysis, and a relatively small sample size, no correction for multiple comparisons was performed.

The experimental protocol of this study has been approved by the IRB committee of the Soroka University Medical Center, #5017. All experiments were performed in accordance with STROBE, the guidelines relevant for epidemiologists and statisticians in conducting observational studies. The informed consent was obtained from all the study participants.

## Supplementary Information

Supplementary Information.

## Data Availability

Data will be provided upon request and following the approval by the IRB approval.
