# Supplementary material for: Exposure to metals and morbidity at eight years follow-up in women of childbearing age
Source: Sci Rep. 2021 Jun 1;11:11429. doi: 10.1038/s41598-021-90904-1 (PMC8169725; doi:10.1038/s41598-021-90904-1)
Supplement: Supplementary file 1 — Supplementary Information. [file 41598_2021_90904_MOESM1_ESM.docx]

**Exposure to metals and morbidity at eight years follow-up in women of childbearing age**

Isabella Karakis, Yael Baumfeld, Daniella Landau, Roni Gat, Nofar Shemesh, Maayan Yitshak-Sade, Ofir Tirosh, Batia Sarov and Lena Novack

**Figure legends in the main article**

**Figure 1:** Metals' burden on morbidity. Results of Weighted Quantile Sum Regression analysis.

Y axis in the graphs shows the list of metals.

R Core Team (2020). R: A language and environment for statistical computing. R Foundation for Statistical Computing, Vienna, Austria. URL https://www.R-project.org/.

**Table 1**. Diagnoses included in the study outcomes

| **Diagnoses** | **ICD code in medical charts** | **Frequency in the study population (N=111 subjects)** |
| --- | --- | --- |
| ***Hematology*** |  |  |
| HEMOGLOBINEMIA | 2832 | 1 |
| LEUKOCYTOSIS | 2888 | 1 |
| LEUKOPENIA | 2880 | 1 |
| NEUTROPHILIA | 2883 | 1 |
| THALASSEMIA MAJOR | 2824 | 1 |
| THALASSEMIA MINOR | 28249 | 1 |
| ***Cancer and benign*** |  |  |
| ANGIOMA | 22800 | 1 |
| BECKER'S NEVUS | 2169 | 2 |
| BENIGN NEOPLASM SKIN | 216 | 6 |
| CAVERNOUS HEMANGIOMA | 228 | 1 |
| HYPERPLASIA ENDOMETRIUM | 6213 | 1 |
| LIPOMA | 214 | 1 |
| LIPOMA OF BREAST | 2141 | 1 |
| MALIGNANT NEOPLASM OF NASOPHARYNX, UNSPECIFIED | 1479 | 2 |
| MALIGNANT NEOPLASM OF OVARY | 1830 | 2 |
| MALIGNANT NEOPLASM OROPHARYNX | 146 | 1 |
| MYXEDEMA PRIMARY NOT SPECIFIED | 2449 | 3 |
| NEOPLASM OF UNCERTAIN BEHAVIOR OF OVARY | 2362 | 1 |
| SECONDARY MALIGNANT NEOPLASM OF RETROPERITONEUM AND PERITONEUM | 1976 | 2 |
| THYROID LUMP;MASS | T15 | 2 |
| ***Cardio-Vascular*** |  |  |
| ACUTE MYOCARDITIS, UNSPECIFIED | 42290 | 2 |
| CHEST PAIN | 7865 | 6 |
| HEART DISEASE, UNSPECIFIED | 4299 | 2 |
| HEMORRHOIDS EXTERNAL | 4553 | 1 |
| MYOCARDITIS TOXIC | 4229 | 1 |
| MYOCARDITIS, UNSPECIFIED | 4290 | 2 |
| PERICARDIAL EFUSSION | 4239 | 1 |
| PHLEBOTHROMBOSIS SEE THROMBOSIS | 451 | 1 |
| PILES | 455 | 3 |
| PRECORDIAL PAIN | 78651 | 2 |
| PULMONARY HYPERTENSION | 4160 | 1 |
| VARICOSE VEINS LEGS | 454 | 7 |
| VASCULAR INSUFFICIENCY | 4471 | 1 |
| UNSPECIFIED CHEST PAIN | 78650 | 1 |
| ***Psychiatric*** |  |  |
| ADJUSTMENT DISORDER | 3099 & F432 | 2 |
| ADJUSTMENT REACTION | 309 | 1 |
| ANTEPARTUM MENTAL DISORDERS OF MOTHER | 64843 | 2 |
| ANXIETY AND DEPRESSION | 3004 | 3 |
| ANXIETY STATE | 3000 & 30000 | 2 |
| DEPRESSION ENDOGENOUS | 2962 | 1 |
| DEPRESSIVE DISORDER | 311 | 7 |
| DEPRESSIVE EPISODE | F32 | 3 |
| EMOTIONAL UPSET | 3080 | 1 |
| OBSESSIVE-COMPULSIVE DISORDER | F42 | 1 |
| SOMNOLENCE | 3074 | 1 |
| UNSPECIFIED NEUROTIC DISORDER | 3009 | 1 |

**Table 1**. Diagnoses included in the study outcomes – cont'

| **Diagnoses** | **ICD code in medical charts** | **Frequency in the study population (N=111 subjects)** |
| --- | --- | --- |
| ***Obesity*** |  |  |
| BMI >30 | 27809 | 3 |
| MORBID OBESITY | 27801 | 2 |
| OBESITY | 27800 | 15 |
| OBESITY (BMI >30) | 28709 & T82 | 21 |
| OBESITY COMPLICATING PREGNANCY, ANTEPARTUM | 64913 | 1 |
| OBESITY COMPLICATING PREGNANCY, WITH DELIVERY | 64911 | 2 |
| OBESITY, UNSPECIFIED | 27800 | 2 |
| OVERWEIGHT (BMI < 30) | 28702 | 2 |
| ***Diabetes Mellitus*** |  |  |
| ABNORMAL GLUCOSE TOLERANCE OF MOTHER, ANTEPARTUM | 64883 | 1 |
| ANTEPARTUM DIABETES MELLITUS | 64803 | 4 |
| DIABETES MELLITUS | 250 | 5 |
| DIABETES MELLITUS IN PREGNANCY | 6480 | 3 |
| DIABETIC ACIDOSIS | 2501 | 1 |
| IMPAIRED FASTING GLUCOSE | 79021 | 1 |
| TYPE II/UNSPECIFIED TYPE, DIABETES MELLITUS WITHOUT COMPLICATION | 25000 | 4 |
| ***Neurological*** |  |  |
| CARPAL TUNNEL SYNDROME | 3540 | 5 |
| CREUTZFELDT-JAKOB DISEASE -CJD | 461 | 27 |
| FACIAL NERVE DISORDERS | 351 | 1 |
| FACIAL PAIN | 7840 | 3 |
| HEADACHE | 7840 | 7 |
| HEADACHE TENSION | 3078 | 4 |
| IMBALANCE | 7812 | 1 |
| LIGHT HEADEDNESS | 7804 | 3 |
| MICTURITION SYNCOPE | 7802 | 1 |
| MIGRAINE | 346 | 4 |
| MIGRAINE, UNSPECIFIED, WITHOUT MENTION OF INTRACTABLE MIGRAINE | 34690 | 1 |
| MYALGIA AND MYOSITIS, UNSPECIFIED | 7291 | 1 |
| PSEUDOINFLAMMATORY FOVEAL DYSTROPHY | 3627 | 1 |
| SHOULDER-HAND SYNDROME | 3379 | 2 |
| TORTICOLLIS SPASMODIC | 3338 | 2 |
| TRANSIENT GLOBAL AMNESIA | 4377 | 1 |
| ***Asthma or other respiratory morbidity*** |  |  |
| ASTHMA ROUTINE FOLLOW UP | V67 | 11 |
| BRONCHIAL ASTHMA | 493 | 1 |
| COUGH | 7862 | 1 |
| DYSPNEA & RESPIRATORY ABNORMALITIES | 7860 | 2 |
| HEMORRHAGE LUNG | 7863 | 1 |
| OTHER DYSPNEA & RESPIRATORY ABNORMALITY | 78609 | 1 |
| OTHER PULMONARY EMBOLISM & INFARCTION | 41519 | 1 |
| PLEURAL EFFUSION UNSPECIFIED | 5119 | 1 |
| WHEEZING | 7860 | 15 |

**Figure 1**: Spearman correlation between metals' concentrations in the study population (n=111 subjects)


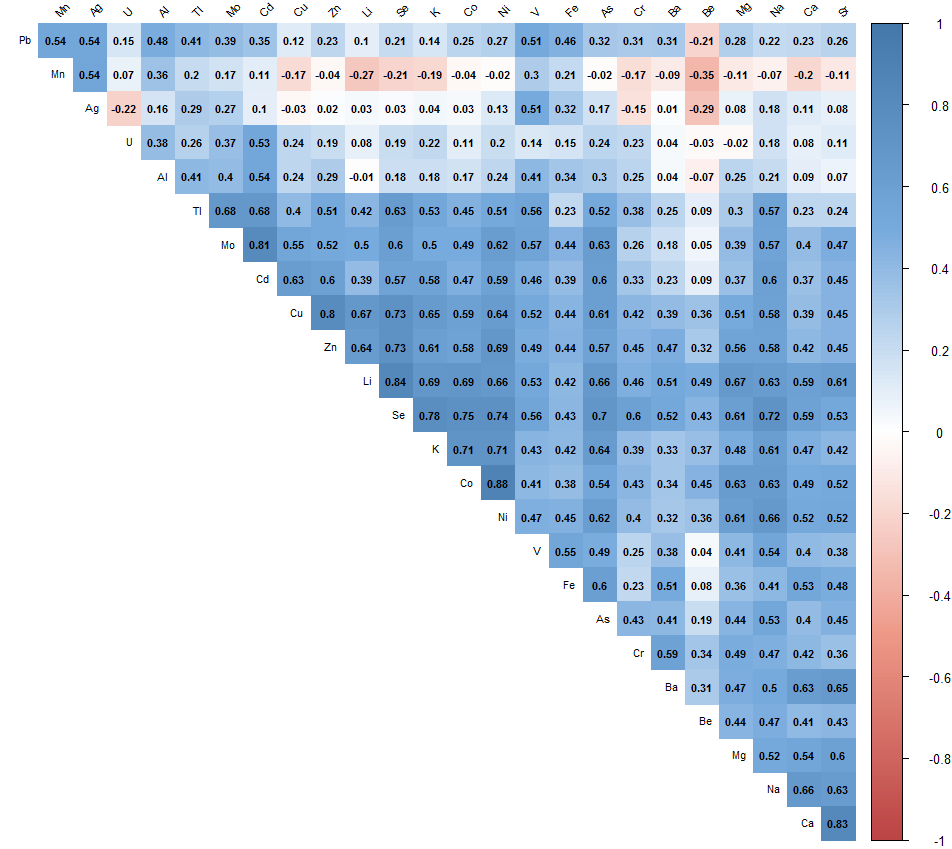


The figure presents only the correlations that were statistically significant (p-value<0.01). All non-significant associations were blanked.

R Core Team (2020). R: A language and environment for statistical computing. R Foundation for Statistical Computing, Vienna, Austria. URL https://www.R-project.org/.
